# Regional differences in advanced gastric cancer: exploratory analyses of the AVAGAST placebo arm

**DOI:** 10.1007/s10120-017-0773-y

**Published:** 2017-10-20

**Authors:** Akira Sawaki, Yasuhide Yamada, Kensei Yamaguchi, Tomohiro Nishina, Toshihiko Doi, Taroh Satoh, Keisho Chin, Narikazu Boku, Yasushi Omuro, Yoshito Komatsu, Yasuo Hamamoto, Wasaburo Koizumi, Shigehira Saji, Manish A. Shah, Eric Van Cutsem, Yoon-Koo Kang, Junko Iwasaki, Hiroshi Kuriki, Wataru Ohtsuka, Atsushi Ohtsu

**Affiliations:** 1 Aichi Cancer Center Hospital, Aichi, Japan; 2 Present Address: National Cancer Center Hospital, Tokyo, Japan; 3 Saitama Cancer Center Hospital, Saitama, Japan; 4 Shikoku Cancer Center, Ehime, Japan; 5 National Cancer Center Hospital East, Kashiwa, Japan; 6 Faculty of Medicine, Kinki University Hospital, Osaka, Japan; 7 Present Address: Cancer Institute Hospital of JFCR, Tokyo, Japan; 8 Shizuoka Cancer Center, Shizuoka, Japan; 9 Tokyo Metropolitan Cancer and Infectious Disease Center, Komagome Hospital, Tokyo, Japan; 10 Hokkaido University Hospital, Sapporo, Japan; 11 School of Medicine, Keio University, Tokyo, Japan; 12 Kitasato University East Hospital, Kanagawa, Japan; 13 International Medical Center, Saitama Medical University, Saitama, Japan; 14 Weill Cornell Medical College/New York Hospital, New York, USA; 15 University Hospitals Gasthuisberg, Leuven and KULeuven, Louvain, Belgium; 16 Asan Medical Center, University of Ulsan, Seoul, South Korea; 17 Chugai Pharmaceutical Co Ltd, Tokyo, Japan; 18 Present Address: Department of Medical Oncology, School of Medicine, Fujita Health University, Toyoake, Aichi 470-1192 Japan; 19 Osaka University Hospital, Osaka, Japan; 20 Present Address: Kitasato University Hospital, Tokyo, Japan; 21 Present Address: Fukushima Medical University, Fukushima, Japan

**Keywords:** Stomach neoplasms, Survival analysis, Geographic locations

## Abstract

**Background:**

AVAGAST was an international, randomized, placebo-controlled phase III study of chemotherapy with or without bevacizumab as first-line therapy for patients with advanced gastric cancer. We performed exploratory analyses to evaluate regional differences observed in the trial.

**Methods:**

Analyses were performed in the placebo plus chemotherapy arm (intention-to-treat population). Chemotherapy was cisplatin 80 mg/m^2^ for six cycles plus capecitabine (1000 mg/m^2^ orally bid days 1–14) or 5-fluorouracil (800 mg/m^2^/day continuous IV infusion days 1–5) every 3 weeks until disease progression or unacceptable toxicity.

**Results:**

Overall, 387 patients were assigned to placebo plus chemotherapy (eastern Europe/South America, *n* = 118; USA/western Europe, *n* = 81; Korea/other Asia, *n* = 94; Japan, *n* = 94). At baseline, poor performance status, liver metastases, and larger tumors were most frequent in eastern Europe/South America and least frequent in Japan. Patients received subsequent chemotherapy after disease progression as follows: eastern Europe/South America (14%); USA/western Europe (37%); Korea/other Asia (61%); and Japan (77%). Hazard ratios for overall survival versus USA/western Europe were 1.47 (95% CI, 1.09–1.99) for eastern Europe/South America, 0.91 (95% CI, 0.67–1.25) for Korea/other Asia, and 0.87 (95% CI, 0.64–1.19) for Japan.

**Conclusions:**

Regional differences in the healthcare environment may have contributed to the differences in overall survival observed in the AVAGAST study.

## Introduction

Gastric cancer is a heterogeneous disease, and its incidence shows wide variation among geographic regions. Although the global incidence of gastric cancer has decreased markedly over the past 40 years, almost 1 million new cases are estimated to have occurred in 2012 [1]. Gastric cancer remains highly prevalent in eastern Asia (mainly in China) and is also common in central and eastern Europe and in central and South America [1]. Western Europe and North America are considered low-risk regions [2], although there has been a steady increase in gastroesophageal junction carcinoma in these geographic regions since the 1970s [3].

AVAstin in GASTric cancer (AVAGAST) was an international, randomised, phase III study designed to compare the efficacy of bevacizumab plus standard chemotherapy versus placebo plus chemotherapy as first-line treatment for patients with advanced gastric cancer [4]. In the primary efficacy analysis, the addition of bevacizumab to chemotherapy significantly prolonged progression-free survival and improved overall response rate, but had no significant benefit on the primary study endpoint, overall survival [4]. Randomization in the AVAGAST trial was stratified by region (Asia Pacific, Europe, pan-America). In pre-planned subgroup analyses, it was evident that efficacy outcomes in both treatment arms varied markedly by region, with patients from the Asia–Pacific region showing no survival benefit from the addition of bevacizumab to chemotherapy, in contrast to patients from other regions [4]. Furthermore, patients from the Asia–Pacific region who received placebo plus chemotherapy had better outcomes than patients from other regions [4].

At the time of the study, regional differences in the prognosis of gastric cancer were one of the major clinical questions. The factors influencing observed differences in prognosis were considered to be either tumor biology or the healthcare environment. The effects of tumor biology on outcomes in the AVAGAST study have already been reported [5, 6], but the impact of the healthcare environment has not been studied. Therefore, we performed a set of exploratory analyses using data from the AVAGAST trial to examine the effect of regional differences in the healthcare environment on study outcomes by investigation of the use of second-line therapy and baseline characteristics. We analyzed data from the placebo group only to exclude the possible effects of bevacizumab. The cutoff date for the primary analysis was November 2009, after a median follow-up of approximately 10 months. The cutoff date for the present analysis was almost 4 years later at the time of trial completion in September 2013.

## Methods

### Study design

AVAGAST was a prospective, randomized, double-blind, placebo-controlled, phase III trial designed to evaluate the efficacy of adding bevacizumab to capecitabine plus cisplatin in the first-line treatment of patients with advanced gastric cancer. The design of AVAGAST has been described in full previously [4]. The protocol was approved at each participating site by an independent ethics committee or institutional review board. The trial was registered (ClinicalTrials.gov identifier: NCT00548548).

### Patients

Patients with histologically confirmed, unresectable, locally advanced, or metastatic adenocarcinoma of the stomach or gastroesophageal junction who had received no previous treatment for metastatic gastric cancer were eligible. Patients could have measurable or non-measurable disease, provided that it was evaluable according to response evaluation criteria in solid tumors (version 1.0) [7]. (Neo)adjuvant chemotherapy was allowed if completed within 6 months before randomization, but previous platinum or anti-angiogenic therapy was not permitted.

### Randomization and treatment

Patients were randomly assigned (1:1) to bevacizumab (Avastin) or placebo plus capecitabine or 5-fluorouracil in combination with cisplatin. Bevacizumab 7.5 mg/kg or placebo was administered as an intravenous infusion on day 1, followed by cisplatin 80 mg/m^2^ as an intravenous infusion, then oral capecitabine 1000 mg/m^2^ twice daily on days 1–14 every 3 weeks. Cisplatin was administered for six cycles; capecitabine and bevacizumab were continued until disease progression or unacceptable toxicity occurred. 5-Fluorouracil (800 mg/m^2^/day as a continuous intravenous infusion on days 1–5 every 3 weeks) was administered instead of capecitabine to patients unable to take oral medications. Treatments given after disease progression were recorded.

### Assessments

Tumor assessments (magnetic resonance imaging and/or computed tomography) were performed within 21 days before randomization and were repeated every 6 weeks for the first year after randomization and every 12 weeks thereafter until disease progression. Survival status was assessed every 3 months after completion of study treatment.

Overall survival, the primary study endpoint, was defined as the time between randomization and death irrespective of cause. Progression-free survival was a secondary endpoint, defined as the time between randomization and first documented disease progression event or death.

### Statistical analysis

The data cutoff date for the primary analysis was November 2009, after 517 overall survival events had occurred [4]. AVAGAST was completed in September 2013; data from this later cutoff were used in the present analysis. All analyses were performed in patients assigned to the placebo plus chemotherapy arm (intention-to-treat population). The present analyses were not previously planned in the study protocol and are exploratory in nature.

Participating countries were divided into four regions: USA and western Europe (Australia, Belgium, France, Germany, Italy, Spain, UK); eastern Europe (Russia) and South America (Brazil, Costa Rica, Peru); Korea and other Asian countries (Korea, China, Singapore, Taiwan); and Japan. Patient baseline characteristics and the proportion of patients who received chemotherapy after disease progression were compared among the four regions. Time-to-event endpoints were analyzed using the Kaplan–Meier method by region. Outcomes for USA/western Europe versus each of the other regions were compared using a Cox proportional hazards model and expressed as hazard ratios with 95% confidence intervals (CIs). A multivariate analysis of overall survival was also performed using Cox proportional hazards regression models with region and other prognostic variables as covariates, and the Wald chi-square test used to test differences between covariates.

## Results

### Patient population

A total of 387 patients were assigned to the placebo plus chemotherapy arm, of which 81 patients were enrolled from USA/western Europe, 118 patients from eastern Europe/South America, 94 patients from Korea and other locations in Asia, and 94 patients from Japan.

Baseline characteristics by region are presented in Table 1. There were some differences between regions with respect to patient and tumor characteristics at baseline. An Eastern Cooperative Oncology Group (ECOG) performance status of 1 or 2, the presence of liver metastases, and a maximum tumor size ≥ 40 mm were observed most frequently in patients enrolled from eastern Europe/South America and least often in patients from Japan. Liver and peritoneal metastases were each present in approximately 30‒40% of patients in patients from USA/western Europe and eastern Europe/South America, whereas in Asian countries liver metastases were documented in 25‒30% of patients and peritoneal metastases in 50‒55% of patients.

**Table 1:** Baseline characteristics by region (placebo plus chemotherapy arm)

Characteristic		USA/western Europe (*n* = 81)	Eastern Europe/South America (*n* = 118)	Korea and other Asia (*n* = 94)	Japan (*n* = 94)
Sex	Male	52 (64)	80 (68)	63 (67)	63 (67)
	Female	29 (36)	38 (32)	31 (33)	31 (33)
Age (years)	< 65	49 (60)	92 (78)	75 (80)	62 (66)
	≥ 65	32 (40)	26 (22)	19 (20)	32 (34)
ECOG performance status	0	42 (52)	28 (24)	36 (38)	62 (66)
	≥ 1	39 (48)	90 (76)	58 (62)	32 (34)
Primary site	Stomach	52 (64)	107 (91)	91 (97)	88 (94)
	Gastroesophageal junction	29 (36)	11 (9)	3 (3)	6 (6)
Disease status	Locally advanced	4 (5)	4 (3)	0	1 (1)
	Metastatic	77 (95)	114 (97)	94 (100)	93 (99)
Liver metastasis	Yes	27 (34)	51 (43)	25 (27)	23 (24)
	No	53 (66)	67 (57)	69 (73)	71 (76)
Number of metastatic sites	> 1	45 (57)	80 (70)	63 (67)	59 (63)
	≤ 1	34 (43)	35 (30)	31 (33)	35 (37)
Histological type^a^	Intestinal	39 (48)	46 (39)	28 (30)	22 (23)
	Diffuse/mixed	40 (49)	68 (58)	52 (55)	72 (77)
Prior gastrectomy	Yes	16 (20)	32 (27)	28 (30)	31 (33)
	No	65 (80)	86 (73)	66 (70)	63 (67)
Prior (neo)adjuvant chemotherapy	Yes	9 (11)	3 (3)	10 (11)	8 (9)
	No	72 (89)	115 (97)	84 (89)	86 (91)
Disease measurability (tumor size)^b^	Not measurable	16 (20)	18 (15)	27 (29)	29 (31)
	Measurable (< 40 mm)	39 (49)	47 (40)	48 (51)	47 (50)
	Measurable (≥ 40 mm)	25 (31)	53 (45)	19 (20)	18 (19)
Peritoneal metastasis	Yes	24 (30)	47 (40)	49 (52)	52 (55)
	No	57 (70)	71 (60)	45 (48)	42 (45)
Bone metastasis	Present	4 (5)	4 (3)	3 (3)	5 (5)
	Not present	76 (95)	114 (97)	91 (97)	89 (95)

Data are expressed as *n* (%)

*ECOG* Eastern Cooperative Oncology Group

^a^Patients with unknown histological type are excluded

^b^One patient from USA/western Europe had a measurable tumour of unknown size

### Post-progression chemotherapy

A summary of chemotherapeutic agents given after disease progression is provided in Table 2. Subsequent chemotherapy after disease progression was given to 17 patients (14%) enrolled from eastern Europe/South America, 30 (37%) from USA/western Europe, 57 (61%) from Korea and other Asia locations, and 72 (77%) from Japan.

**Table 2:** Post-progression chemotherapy for gastric cancer (placebo plus chemotherapy arm)

Treatment	USA/western Europe (*n* = 81)	Eastern Europe/South America (*n* = 118)	Korea and other Asia (*n* = 94)	Japan (*n* = 94)
Total	30 (37)	17 (14)	57 (61)	72 (77)
Paclitaxel	3	2	15	52
Docetaxel	12	2	24	13
Irinotecan	10	7	22	32
5-Fluorouracil	14	6	32	18
S-1	0	0	9	14
Capecitabine	4	2	2	0
Methotrexate	0	0	2	12
Folinic acid	8	5	26	5
Cisplatin	2	3	10	7
Oxaliplatin	2	2	17	3

Data are expressed as *n* (%) or *n*

### Overall survival and progression-free survival

**Fig. 1:**
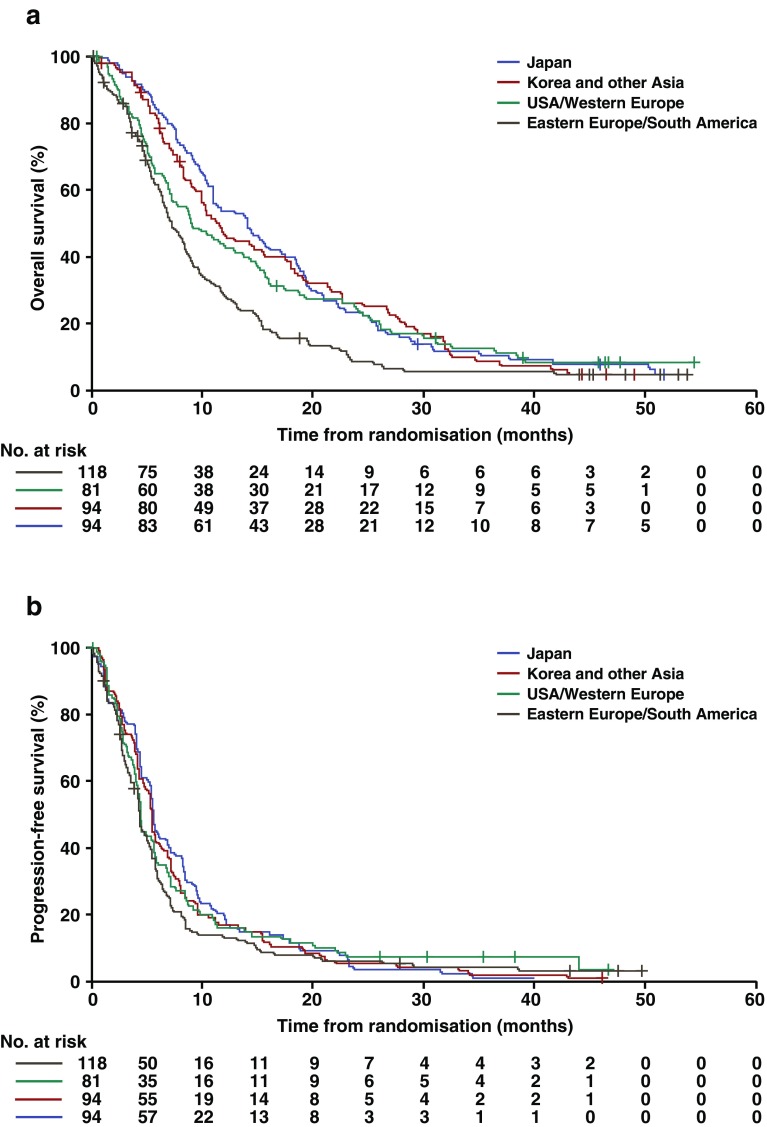
Kaplan–Meier curves by region of (**a**) overall survival and (**b**) progression-free survival. Data are for the placebo plus chemotherapy arm only

At the cutoff date, the median duration of follow-up in the placebo plus chemotherapy arm was 9.7 months (range, 0.1–54.5 months). A summary of efficacy by region is presented in Table 3. In the placebo plus chemotherapy arm, a total of 348 patients (90%) had died. The median duration of overall survival was 7.3 months (95% CI, 6.4–8.7) in eastern Europe/South America, 9.1 months (95% CI, 6.9–14.4) in USA/western Europe, 11.6 months (95% CI, 9.1–15.6) in Korea and other Asia, and 14.1 months (95% CI, 10.9–17.6) in Japan. Kaplan–Meier curves for overall survival showed that the prognosis of patients in eastern Europe/South America tended to be worse than in other regions (Fig. 1a). The hazard ratios for overall survival for each region when compared against USA/western Europe were 1.47 (95% CI, 1.09–1.99) for eastern Europe/South America, 0.91 (95% CI, 0.67–1.25) for Korea and other Asia, and 0.87 (95% CI, 0.64–1.19) for Japan. After adjusting for key prognostic factors (Table 4), the hazard ratios for overall survival for each region when compared against USA/western Europe showed little change [1.52 (95% CI, 1.08–2.14) for eastern Europe/South America, 0.82 (95% CI, 0.57–1.18) for Korea and other Asia, and 0.81 (95% CI, 0.57–1.14) for Japan].

**Table 3:** Efficacy outcomes by region (placebo plus chemotherapy arm)

Region	Number of events	Median, months (95% CI)	Hazard ratio^a^ (95% CI)
Overall survival			
USA/western Europe (*n* = 81)	72	9.1 (6.9–14.4)	–
Eastern Europe/South America (*n* = 118)	105	7.3 (6.4–8.7)	1.47 (1.09–1.99)
Korea and other Asia (*n* = 94)	84	11.6 (9.1–15.6)	0.91 (0.67–1.25)
Japan (*n* = 94)	87	14.1 (10.9–17.6)	0.87 (0.64–1.19)
Progression-free survival			
USA/western Europe (*n* = 81)	75	4.4 (4.0–5.7)	–
Eastern Europe/South America (*n* = 118)	111	4.4 (4.0–5.4)	1.19 (0.89–1.59)
Korea and other Asia (*n* = 94)	93	5.6 (4.8–6.5)	1.01 (0.74–1.37)
Japan (*n* = 94)	92	5.7 (5.1–7.0)	0.96 (0.71–1.31)

*CI* confidence interval

^a^Compared with USA/western Europe

**Table 4:** Cox proportional hazards analysis of overall survival (placebo plus chemotherapy arm)

Covariate	Hazard ratio (95% CI)	*p* value
Japan vs. USA/western Europe	0.81 (0.57–1.14)	0.2270
Korea and other Asia vs. USA/western Europe	0.82 (0.57–1.18)	0.2936
Eastern Europe/South America vs. USA/western Europe	1.52 (1.10–2.14)	0.0170
Sex (female vs. male)	1.01 (0.80–1.28)	0.9275
Age (< 65 vs. ≥ 65 years)	0.90 (0.70–1.16)	0.4050
ECOG performance status (0 vs. ≥ 1)	0.73 (0.57–0.93)	0.0116
Primary site (gastroesophageal junction vs. stomach)	1.11 (0.77–1.60)	0.5754
Disease status (locally advanced vs. metastatic)	2.81 (1.33–5.92)	0.0067
Liver metastases (no vs. yes)	0.71 (0.53–0.94)	0.0161
Histological type (diffuse vs. mixed)	0.98 (0.62–1.57)	0.9434
Histological type (intestinal vs. mixed)	0.75 (0.47–1.22)	0.2473
Prior gastrectomy (no vs. yes)	1.61 (1.23–2.11)	0.0006
Prior (neo)adjuvant therapy (no vs. yes)	0.74 (0.49–1.12)	0.1510
Tumor size (< 40 mm vs. not measurable)	1.17 (0.86–1.58)	0.3251
Tumor size (≥ 40 mm vs. not measurable)	1.04 (0.74–1.46)	0.8259
Bone metastases (no vs. yes)	0.45 (0.25–0.78)	0.0050
Peritoneal metastases (no vs. yes)	0.55 (0.42–0.73)	< 0.0001
Number of metastatic sites (> 1 vs. ≤ 1)	0.96 (0.73–1.26)	0.7584

*ECOG* Eastern Cooperative Oncology Group

In the placebo plus chemotherapy arm, a total of 371 patients (96%) had progression-free survival events. Median progression-free survival by region was 4.4 months (95% CI, 4.0–5.4) in eastern Europe/South America, 4.4 months (95% CI, 4.0–5.7) in USA/western Europe, 5.6 months (95% CI, 4.8–6.5) in Korea and other Asia, and 5.7 months (95% CI, 5.1–7.0) in Japan. Kaplan–Meier curves of progression-free survival by region indicated that the prognosis of patients in eastern Europe/South America tended to be worse than in other regions (Fig. 1b). The hazard ratios for progression-free survival for each region compared against USA/western Europe were 1.19 (95% CI, 0.89–1.59) for eastern Europe/South America, 1.01 (95% CI, 0.74–1.37) for Korea, and other Asia and 0.96 (95% CI, 0.71–1.31) for Japan.

## Discussion

The AVAGAST trial was designed to investigate the efficacy of adding a targeted agent—bevacizumab—to standard first-line chemotherapy for patients with advanced gastric cancer. Pre-planned subgroup analyses revealed marked differences between regions in overall survival, estimates that were viewed as robust because 60–70% of patients had died at the time of analysis [4]. The present set of exploratory analyses was performed to better understand the reasons for the regional differences observed in the AVAGAST study.

In our analyses, we observed that patients enrolled in eastern Europe/South America had the shortest median overall survival (7.3 months), followed by patients from USA/western Europe (9.1 months), with patients from both Asian regions showing the longest overall survival times (11.6–14.1 months). The same regional ranking was apparent in other study parameters, i.e. some baseline characteristics and the use of post-progression chemotherapy. We suggest that these differences may have contributed to the regional differences in survival outcomes.

In AVAGAST, the use of further lines of chemotherapy after disease progression was lowest in eastern Europe/South America (14% of patients), followed by USA/western Europe (37%), with the highest usage evident in Korea (61%) and Japan (77%). The inverse relationship between the use of post-progression chemotherapy and overall survival suggests that this was a likely contributing factor to the different survival outcomes between regions. Similar observations have been made in the RAINBOW trial, where uptake of chemotherapy after discontinuation of study treatment was also higher in patients enrolled from Asian centers (67% vs. 37% of patients from other regions), along with improved overall survival (median, 10.5 months in Asia vs. 5.9 months in other regions) [8].

A possible reason for the differential uptake of second-line therapy is that there was no clear evidence to support the use of chemotherapy after disease progression yet there was considerable risk of toxicity. In the intervening years, several phase III randomised trials have demonstrated that second-line chemotherapeutic [9–12] or targeted agents [8, 13] significantly improve overall survival in patients with gastroesophageal cancer compared with best supportive care/active symptom control. These trials have established the role of second-line therapy in patients with gastroesophageal cancer, a practice that is now endorsed in major European [14] and US [15] clinical practice guidelines. Other possible reasons to explain the differential use of post-progression chemotherapy may include differences in health insurance between countries. Few drugs are reimbursed for second and later lines of therapy in patients with gastric cancer in eastern Europe and South America, whereas more drugs are reimbursed in Japan. Not all patients are physically fit enough to be offered second-line therapy. In our analysis of patients’ baseline characteristics, the likelihood of a poor ECOG performance status was greatest in eastern Europe/South America and lowest in the Asian regions, which may also have influenced the choice of therapy.

We also identified differences in baseline tumor characteristics among regions. Patients in eastern Europe/South America were more likely to have liver metastases and measurable disease ≥ 40 mm at baseline than patients enrolled from other regions; patients from both Asian regions had the lowest rates for both parameters. Conversely, peritoneally limited disease was more common in Asian countries. These findings are consistent with an earlier diagnosis of gastric cancer and are probably linked to the screening programs that are in place in some Asian countries. Japan and Korea have ongoing nationwide screening programs, both of which were in place before AVAGAST started [16–19]. Further, Taiwan has a screening program for a high-risk population (on Matzu Island) [18, 19]. A secondary prevention program (i.e., upper gastrointestinal endoscopy for all symptomatic cases) in patients aged ≥ 40 years was also established in Chile in 2006 [20]. Other countries and regions with a lower risk of gastric cancer do not have screening programs because they are not likely to be cost effective. In our analysis, earlier detection of primary tumors in countries with screening programs may have led to earlier identification of metastatic disease and an apparently longer overall survival (i.e., lead-time bias) versus regions without screening programs.

Molecular heterogeneity may also underpin the findings from our study, although gene expression profiling was not performed in AVAGAST. The Cancer Genome Atlas Research Network reported differences in pathway-level gene expression between patients from eastern Asia compared with patients from other regions [21]. Another recent study revealed that tumors from Asian and non-Asian patients exhibited distinct differences in gene signatures related to inflammation and immunity, with T-cell pathways being preferentially associated with gastric cancers in non-Asian patients compared with Asian patients [22]. Further, the Asian Cancer Research Group used gene expression data to identify four distinct molecular subtypes that were associated with disease progression and prognosis [23]. Notably, the proportions of patients with these tumor subtypes varied according to geographic region [23]. It is likely that such studies will provide further insight into tumor characteristics and their implications for prognosis.

It is interesting to note that the overall survival of patients with advanced gastric cancer in clinical trials performed in the 1990s was relatively similar among regions (Table 5). It seems unlikely that major changes in biological characteristics of gastric tumors have developed in the past decade to explain the regional differences in AVAGAST. Rather, we suggest that differential changes in clinical practice (i.e., improved early diagnosis in high-risk countries, use of second-line therapy) may have had a greater effect than biological differences. It is also interesting to note that data from more recent studies (Table 5) show gradual improvements in overall survival in non-Asian regions and a trend toward convergence of survival times.

**Table 5:** Overall survival in phase III trials of first-line platinum/fluoropyrimidine-based regimens in advanced gastric cancer

References	Region/country	Enrollment period	Treatment	No. of patients	Median overall survival, months
Kim et al. [24]	Korea	1986–1990	FP	103	8.5
Vanhoefer et al. [25]	Europe	1991–1995	FP	134	7.2
Ohtsu et al. [26]	Japan	1992–1997	FP	105	7.1
Van Cutsem et al. [27]	International	1999–2003	FP	230	8.6
Dank et al. [28]	International	2000–2002	FP	165	8.7
Cunningham et al. [29]	UK/Australia	2000–2005	EPF	263	9.9
			EPC	250	9.9
			EOF	245	9.3
			EOC	244	11.2
Koizumi et al. [30]	Japan	2002–2004	SP	148	13.0
Kang et al. [31]	International	2003–2005	FP	156	8.9
			CP	160	10.4
Ajani et al. [32]	USA/Europe/South America	2005–2007	SP	527	8.6
			FP	526	7.9
Ohtsu et al. [4]	International	2007–2008	CP	387	10.1
Lordick et al. [33]	International	2008–2010	CP	455	10.7
Waddell et al. [34]	UK	2008–2011	EOC	275	11.3
Kim et al. [35]	Korea	2009–2012	CP	124	11.5
Shen et al. [36]	China	2009–2010	CP	102	11.4
Yamada et al. [37]	Japan	2010–2011	SP	324	13.1
			SO	318	14.1

Only data for the platinum/fluoropyrimidine-containing study arms are shown

*C* capecitabine, *E* epirubicin, *F* 5-fluorouracil, *O* oxaliplatin, *P* cisplatin, *S* S-1

The analyses reported in the present paper were exploratory in nature and hypothesis generating only but may assist with the design of future international trials in advanced gastric cancer. We believe that there are no regional differences in drug efficacy in general. If regional differences related to other factors exist, they should be considered and kept to a minimum by appropriate study design, not only with regard to stratification factors but also with the statistical design based on the assumed overall survival of the patient population included in the study.

In conclusion, regional differences in the healthcare environment (i.e., use of second-line chemotherapy and screening programs) may have contributed to the differences in prognosis observed in the AVAGAST study. As second-line therapy for metastatic disease and screening programs in high-risk populations become more widely used, differences in outcomes in patients with advanced gastric cancer may diminish among regions.
